# The Role of Social Media in Sports Vision

**DOI:** 10.3390/ijerph18105354

**Published:** 2021-05-18

**Authors:** Henrique Nascimento, Clara Martinez-Perez, Cristina Alvarez-Peregrina, Miguel Ángel Sánchez-Tena

**Affiliations:** 1 ISEC LISBOA—Instituto Superior de Educação e Ciências, 1750-179 Lisboa, Portugal; henrique.nascimento@iseclisboa.pt (H.N.); masancheztena@ucm.es (M.Á.S.-T.); 2 Faculty of Sport Sciences, Universidad Europea de Madrid, 28670 Madrid, Spain; 3 School of Biomedical and Health Science, Universidad Europea de Madrid, 28670 Madrid, Spain; cristina.alvarez@universidadeuropea.es; 4 Department of Optometry and Vision, Faculty of Optics and Optometry, Universidad Complutense de Madrid, 28037 Madrid, Spain

**Keywords:** sport vision, social media, social network analysis, Altmetric

## Abstract

Background: Sports vision is a relatively new specialty. The objective is to provide ophthalmological and optometric care services for the care of vision in the sports field. An increasing number of athletes and coaches are trying to improve visual skills and they seek information on social media. The current excess of information has made it increasingly difficult to identify high quality articles. For this reason, alternative metrics are useful tools to identify publications that draw attention to society. This research aims to study the influence of social networks on the importance of vision in sport. Methods: Altmetric Explorer was used to perform a search using “sport”, “vision” and “eye” as keywords. The 100 outcomes with the most attention were analyzed and correlated with the number of citations in the Web of Science (WoS) using the Spearman correlation coefficient. Results: The 100 best Altmetric Attention Scores (AASs) were published in 67 journals and had a mean AAS value of 30.22 ± 62.37 The results were discussed mainly on Twitter, with a mean of 113.99 ± 43.86 tweets and retweets and a mean of 75.92 ± 79.92 readers in Mendeley. There was no correlation between AAS and WoS Cites for the top 100 outcomes and the correlation was low if we considered the total research results rather than the top 100. Conclusions: The citations are not related to the impact of scientific articles on social networks. Sports vision is a specialty with a growing interest in social media.

## 1. Introduction

Nowadays, sports and media share a symbiotic relationship in which both exert an inexhaustible and continuous influence. The media generates revenue through sport while sports and related contents are transmitted through the media in order to develop and expand [1]. This is associated with the fact that many people around the world, especially the younger generation, take part in debates on Facebook, tweet or like a picture on a daily basis [2]. In this regard, sports clubs use advanced and effective communication tools, creating a positive image through social media with the aim of reaching out to people [3]. 

On the other hand, competition in sport relies on athletes having a diverse set of physical and mental skills. Athletes and coaches are constantly looking for ways to improve these skills and given the demanding nature of sports with regard to perception, visual and motor skills are often the foci of sports training programs. Consequently, social media play an increasingly important role in showing ever-improving skills to action sports participants and in contributing to the creation of new links between corporations, action sports organizations and communities [4,5]. These new links have increased the visibility of the specialty of “sports vision” in the field of optometry. The aim of this specialty is to improve and preserve visual functions for better sporting performance. Thus, in the last 10 years, the number of publications on this subject has increased by 30% due to the great interest of both coaches and athletes in improving visual skills and performance on the playing field. Since 2011, the number of publications has increased significantly (1911–2010: 34.96% of publications; 2011–2020: 65.04% of publications). 2019 was the year with the highest number of publications [6,7,8,9,10,11].

For many years, the Journal Impact Factor (JIF) was the best tool to determine a journal’s impact. Nowadays, social networks such as Mendeley or Twitter offer data (known as alternative metrics or Altmetrics), which allow the measurement of the impact of research in a broader way than by means of citations [12]. The term “Altmetrics” was coined by Jason Priem in 2010 to help researchers filter information and identify relevant sources. Originally, this term was used to refer to the metrics derived from social media activity and other alternative sources of information that transcend the scientific field [13]. However, it is now used with very different metrics and sources that combine with each other [14]. This is particularly important in research where several scholars have found limitations in traditional bibliometric measurements because the information displayed on social media is targeted at a large population [15,16].

Altmetric Explorer tracks the attention that research findings such as academic papers and datasets draw online. In other words, Altmetrics provides an overview of how research is shared and discussed online including by audience [17]. This is highly relevant to sports vision as more and more athletes, coaches, physical trainers and teams are seeking information about training techniques to improve their visual skills. 

To date, many studies have analyzed the top Altmetric articles in various areas of health. However, the top Altmetric articles on the influence of vision in sport have not been analyzed. This research aims to study the influence of social networks on the importance of vision in sport.

## 2. Materials and Methods

### 2.1. Database

The search was conducted using Altmetric Explorer (Altmetric LLP, London, UK). The search was performed on 5 April 2021 using “sport”, “vision” and “eye” as keywords. The search field for the first two keywords was Subject Area (applying filters for abstract, title and keywords) and for the third keyword, a query was used that included all fields. All publications resulting from this search were included in the study. The top 100 with the highest attention according to the Altmetric Attention Score (AAS) were analyzed by two researchers who excluded irrelevant results. For the selection of the most mentioned articles, the AAS provided by Altmetric.com was chosen. The data provided by Altmetric are the most comprehensive, covering the vast majority of social media activity associated with scientific articles. The AAS reflects a weighted total of the mentions of the article by the different online platforms. Thus, a news item is worth eight points, a tweet is worth one point and a Facebook post is worth a quarter point [18]. To perform the analysis, the number of articles with the highest AAS was established as the average of the most cited and most downloaded articles of each journal (after rounding the average number of each journal). The articles with an AAS mat were then identified by an Altmetric Explorer search (Altmetric LLP, London, UK).

### 2.2. Data Analysis

The Web of Science (WoS) was used to obtain the number of citations for each result, which was then compared with the AAS for the same search date.

The correlation between the number of citations on the WoS and the publication data obtained with the AAS was tested using the Spearman correlation coefficient with SPSS software (IBM, Armonk, NY, USA).

## 3. Results

The Altmetric Explorer search provided 157 research outputs out of 234 published according to the Web of Science (WoS). A total of 201 of these outputs were mentioned at least once, with a total of 2993 mentions. The first output was from 1995, with one mention in video databases. The years 2017 and 2020 had the highest number of outputs (*n* = 26) followed by 2018 (*n* = 23) and 2019 (*n* = 21). There was a low correlation between publication date and the AAS (Altmetric Attention Score) (r = 0.145; *p* = 0.028). Table 1 shows the mean and standard deviation of mentions since 2007 and the total number of mentions in any of the sources studied according to the year as that is when the first iPhone appeared and social networks began to have relevance. 

Twitter was the source with the most mentions with the USA (769 posts and 556 profiles), the UK (666 posts and 344 profiles) and Australia (220 posts and 169 profiles) as the three most active countries sharing information on this social network. A total of 769 posts and 556 profiles were not related to any country (Table 2).

The research outputs from the top 100 AASs were published in 67 journals and had a mean AAS value of 30.22 ± 62.37 (range 3 to 426). The outputs were mainly discussed on Twitter with a mean of 13.99 ± 43.86 tweets and retweets (range 0 to 484). Supplementary Figure S1 shows the mentions on social networks of the publications with the most AASs as well as the number of readers in Mendeley.

Regarding the journals, *Optometry and Vision Science* was the journal with the highest number of articles in the top 100 with a total of five papers. *Journal of Science and Medicine in Sport*, *JAMA Ophthalmology* and *Journal of the Neurological Sciences* fell behind *Optometry and Vision Science* with three articles each among the top 100. Table 3 and Table 4 show the characteristics of the ten journals with the highest AAS on vision and sport. Thus, during the years included in the study, 23 out of 51 items published had an AAS higher than 1 and, of these, five had an AAS higher than 5. *JAMA Ophthalmology* had the highest cumulative AAS. The journals with the highest attraction were *JAMA Ophthalmology* and *Medicine & Science in Sports & Exercise* with a mean AAS per published item of 131.3 and 75.5, respectively. *Optometry and Vision Science* drew online attention to 61.5% of its published articles and this journal published the highest number of items with an AAS above 5.

In terms of Field of Research (FoR) of the top 100 outputs, 75 were classified in division 11: Medical and Health Sciences and 23 of them in division 17: Psychology and Cognitive Sciences. The main area of the journals in which it was published was Medical and Health Sciences with 74 of the 100 outputs studied. Regarding the main areas of the reviews in which sports vision articles appeared, we found Neurosciences with 19 of the 100 outputs and Human Movement and Sports Science with 16 of the 100 outputs.

Table 5 shows the five outputs with the highest AAS as well as other traditional bibliometric parameters.

When studying the correlation between the AAS and WoS Cites, no correlation was found between both values for the top 100 outputs (*r* = 0.195; *p* > 0.05). However, a low correlation was found when analyzing the total outputs (*r* = 0.147; *p* = 0.024).

## 4. Discussion

Visual skills are essential for most sports because visual information can account for up to 85–95% of the sensory information an athlete receives on the playing field. This is why sports vision is now a fundamental part of athlete development and daily training has the potential to enhance performance [19,20]. The study by Spera et al. [21] showed that athletes with a visual impairment had less chance of winning a competition. This is because the functions of the loss of vision affects movement coordination, balance and emotional state, which are important particularly for martial arts. Another study showed that good vision is important for the athlete and training will allow a quick response to different types of stimuli [22].

In recent years, a growing interest on the part of trainers and athletes has led to an increase in posts about this area of research on social media and how to try to improve visual skills for better results on the playing field. As a consequence, it has also become increasingly important to know how the population is receiving this information.

In this regard, altmetrics covers social media activity in the form of mentions on social media, academic activity in digital libraries, popularity indexes in reference managers, scholarly comments through scientific blogs and references on social media [23].

This study is relevant in terms of understanding the impact of sports vision research on society. Compared with other subject areas, sports vision seems to be a topic that currently receives little attention, with a mean AAS value of 30.22 in the top 100 research outputs. This may be due to the fact that the number of publications on sports vision has increased since 2011, which means that it is a new specialty in the field of optometry [6]. However, it is important to note that only 34 of the top 100 research outputs were published in optometry, ophthalmology or sports journals. The top two research outputs according to the AAS were published in *PLoS ONE* [24] and *JAMA Ophthalmology* [25], with 500 and 117 mentions and 37 and 16 citations on the WoS, respectively. In third position was the publication by Master et al. in 2018 in the *Clinical Journal of Sport Medicine* [26] with 111 mentions and 37 citations on the Web of Science. This difference may be due to the fact that this research area is multi-disciplinary and researchers in this field tend to be published in journals with a wide range of research topics.

Analyzing the five most relevant papers in terms of the AAS, the article by Saphiro et al. [24] entitled “Transitions between central and peripheral vision create spatial/temporal distortions: a hypothesis concerning the perceived break of the curveball” was first. It describes the perception of the ball in baseball. In terms of metrics, it could be observed that the largest number of mentions occurred on Twitter although it could also be found on other social networks. This could be explained by the fact that this is one of the most popular sports in the United States and the news was published in The Washington Post newspaper, which gave it a very high visibility. The article analyses the surprise at the explanation offered by science of the perception the batter has of the ball when he tries to hit it as the visual perception does not match the reality, thus worsening the outcome in batting. The relevance of this analysis should not be disregarded as it might make a certain team win matches and even championships if they can count on a good pitcher who is able to give enough spin to the ball so as to cause an erroneous visual perception. The news became popular and was echoed on Twitter where the hashtag “the curveball illusion” caught the attention of readers and helped in its dissemination. It was a blend of science, sport and perception.

In second position was the article by Haring et al. [25] entitled “Epidemiology of Sports-Related Eye Injuries in the United States”, which provided an annual incidence of sports-related eye trauma broken down by age, sex, the mechanism of the injury and related activity as well as factors associated with short-term vision problems. As a result, they found that, in the United States, about 70% of eye injuries are sports-related. Again, baseball and basketball appear as the sports with the highest incidence of eye injuries. As these are the two major sports in the United States, they are more widespread and relevant. In relation to this article, the importance of eye protection plans in sport was highlighted. In this case, it was the magazine itself that first spread the article through Twitter. Scientific journals taking part in this type of dissemination is a relatively new practice, which highlights the inclusion of social networks in science outreach plans.

In position 3 and 5 of the most relevant papers were those on American football and the head concussions that occur in the practice of this sport. It is a sport with a high incidence of concussion during its practice. As can be seen in article [27], a total of 1302 concussions were analyzed between 2015 and 2019 in 1004 players, of which 80% of the cases refer to football within the National Football League (NFL) professional league in the United States. Again, this is another highly popular sport in this country.

These papers analyze the repercussions that this type of concussion can have on children’s development.

In this instance, the tweets came from the medical area, from medical professionals with many followers disseminating this scientific knowledge and expressing their concern.

In fourth position we have to highlight the study by Aksum et al. [28] published in October 2020. Even if it did not have any citations on the WoS, it was one of the five papers that drew the most attention on social media. This indicated the importance of evaluating alternative metrics that give faster information than traditional metrics about the interest in a certain research project. The attraction of this paper was due to the fact that it analyses the importance of vision on the playing field in elite soccer players and, as mentioned above, a greater number of coaches and athletes are looking to improve their visual skills.

This article refers to soccer, a major sport in Europe and Asia as proven by the fact that most of the tweets came from Sweden and China.

With regard to the years, 2010 and 2017 were the years where publications drew the highest attraction with 540 and 421 mentions, respectively. In those years, the papers by Shapiro et al. [24] and Gallaway et al [29] are worth mentioning. However, when we compared altmetric studies with bibliometric studies, the year 2019 stood out due to the large number of publications and great progress in the research field [6].

This difference may be due to the fact that in 2010, three major multi-sport events took place, the Winter Olympic Games, the FIFA World Cup and the Commonwealth Games. Thus, in countries such as Spain, during that year, interest in sport increased as this country won the World Cup, Alberto Contador, a Spanish cyclist, won the Tour de France and Rafael Nadal, a Spanish tennis player, took over from Roger Federer as a prominent figure in the world of tennis. During 2017, a historic Super Bowl final took place and sprinter Usain Bolt ran his last international races. All this led to increased social interest in the world of sport. On the other hand, in terms of the number of scientific publications, 2019 was the most relevant year, most definitely due to the run up to the Tokyo 2020 Olympic Games. Unfortunately, it is not possible to confirm whether these publications really drew more attention because of the one-year suspension of the Olympic Games due to the pandemic.

In terms of sources, Twitter was the most widely used with the United States and the United Kingdom leading in profiles and tweets on sports vision research. This was consistent with the US being the country with the largest number of users (59,350,000 users) and both the UK and the US being English-speaking countries, which gives rise to a possible connection between the different research groups. At the same time, it was observed that the most prominent articles in this rank referred to three priority sports in the United States: basketball, baseball and American football.

However, if compared with other studies such as the one conducted by Kharmalki et al. [30], Instagram was found to entice a higher participation in the area of sport in general compared with Facebook and YouTube. This may be due to the fact that fans currently use Instagram more to interact with their sports team compared with Facebook, Twitter or YouTube as they prefer instant viewing. In addition, there is growing evidence that fans have moved away from the traditional way of consuming sports where there is only one-way communication and are more drawn to social networking sites where communication is instant and they are connected to a two-way communication network.

As a result, it is expected that in the near future, given the growing interest in the influence of vision on sports performance, public engagement on social media will increase. It should be noted that despite the growing interest in training visual skills to improve athletic performance, it is still unknown how visual training will improve performance on the field of play. Therefore, the increased interest in social networks about the influence of vision in sport will help to develop various training programs in the future to continue training the most relevant visual skills for sport in order to improve performance on the field of play. In addition, it is also hoped that sports vision techniques can be used to assess and rehabilitate sports-related concussions.

The number of publications has grown but it is yet to be seen how it will influence the interest shown on social media.

## 5. Conclusions

Social networks play an important role for sport vision.

This study offered a new insight into the use and impact of online research given the increase of open access publications. In addition, it showed that Twitter was the social media network with the highest number of mentions of scientific articles in the area of sports vision, with the United States being the country with the highest number of mentions.

The more powerful social networks are to publish the news, the more it can lead to a greater reach and credibility of the investigation.

In this sense, American football is the sport that presented the most interest on social media in which Twitter stood out followed by Facebook. The article with the greatest attraction was published in *PLoS ONE*. Therefore, it should be noted that the citations were not related to the impact on social networks of the scientific article as the most published journal on social media is *Optometry and Vision Science*.

## Figures and Tables

**Table 1:** Mean and standard deviation of mentions by source and total number of mentions that Altmetric Explorer tracked by sport and vision according to year.

	Mean ± SD	2007	2008	2009	2010	2011	2012	2013	2014	2015	2016	2017	2018	2019	2020	2021
News	12.6 ± 17.3	0	0	0	9	3	5	15	7	8	63	38	23	13	5	0
Blog	2.4 ± 3.2	0	0	0	12	5	2	6	0	3	2	1	1	3	1	0
Policy *	0.4 ± 0.7	0	0	0	1	0	1	2	0	0	2	0	0	0	0	0
Patent **	1.3 ± 2.9	0	0	0	1	0	9	8	0	2	0	0	0	0	0	0
Twitter	162.3 ± 160.7	0	5	2	505	22	37	254	20	185	243	339	289	187	329	18
Peer review **	0.1 ± 0.3	0	0	0	0	0	0	1	0	1	0	0	0	0	0	0
Facebook	12.2 ± 13.5	0	0	0	10	3	17	27	5	33	23	41	18	3	3	0
Wikipedia	1.3 ± 1.5	0	0	1	1	2	3	3	0	5	0	0	0	2	2	0
Google †	1.3 ± 2.3	0	0	0	1	9	1	0	0	0	3	1	2	2	0	0
Reddit ***	0.1 ± 0.2	0	0	0	0	0	0	0	0	0	0	1	0	0	0	0
Video ††	0.1 ± 0.2	0	0	0	0	1	0	0	0	0	0	0	0	0	0	0
Total	194.1 ± 179.8	0	5	3	540	45	75	316	32	237	336	421	333	210	340	18

* not trackable since 2016; ** not trackable since 2015; *** not trackable since 2017; † not trackable since 1999; †† not trackable since 2011.

**Table 2:** Mention of the journals on the different social media platforms.

Journal Title	News	Blog	Twitter	Facebook	Wikipedia	Google	Video
*Optometry and Vision Science*	41	2	107	32	0	0	1
*JAMA Ophthalmology*	75	6	84	26	0	7	0
*Journal of the Neurological Sciences*	0	0	122	5	2	0	0
*Medicine & Science in Sports & Exercise*	11	3	59	11	0	0	0
*Journal of Science and Medicine in Sport*	0	0	49	0	2	0	0
*Journal of Pediatric Ophthalmology and Strabismus*	1	0	19	1	5	1	0
*Experimental Brain Research*	0	0	37	1	0	0	0
*Journal of Sports Sciences*	1	0	64	4	1	0	0
*European Journal of Sports Sciences*	0	0	24	0	0	0	0
*Frontiers in Psychology*	0	0	227	1	0	0	0
Total	129	11	792	81	10	8	1

**Table 3:** Journals in the top 10 Altmetric Attention Score in sport vision research.

Journal Title	*n*	Number of Mentioned Outputs	Total Mentions	AAS	IF	Citations, WoS
*Optometry and Vision Science*	13	10	180	371	8.470	186
*JAMA Ophthalmology*	4	4	198	525	6.198	68
*Journal of the* *Neurological Sciences*	7	6	141	57	3.115	357
*Medicine and Science in* *Sports & Exercise*	2	2	84	151	4.029	25
*Journal of Science and* *Medicine in Sport*	4	3	49	28	3.607	26
*Journal of Pediatric Ophthalmology and Strabismus*	3	2	15	26	1.100	85
*Experimental Brain Research*	2	2	38	21	1.591	16
*Journal of Sports Sciences*	8	4	58	32	2.597	152
*European Journal of Sports Sciences*	3	3	24	12	2.781	11
*Frontiers in Psychology*	5	4	226	7	2.067	47
Total	51			1230		973

*n*: Number of published items; AAS: Altmetric Attention Score.

**Table 4:** AAS rank for each of the journals studied.

Journal Title	AAS/Article	*n*/AAS	*n*/AAS Range			
			1	2–5	6–10	>10
*Optometry and Vision Science*	28.5	8 (61.5%)	2	2	1	3
*JAMA Ophthalmology*	131.3	0 (0%)	0	0	0	0
*Journal of the Neurological Sciences*	8.1	3 (42.8%)	2	1	0	0
*Medicine & Science in Sports & Exercise*	75.5	0 (0%)	0	0	0	0
*Journal of Science and Medicine in Sport*	7.0	2 (50%)	1	1	0	0
*Journal of Pediatric Ophthalmology and Strabismus*	8.7	0 (0%)	0	0	0	0
*Experimental Brain Research*	10.5	0 (0%)	0	0	0	0
*Journal of Sports Sciences*	4.0	4 (50%)	2	1	1	0
*European Journal of Sports Sciences*	4.0	2 (66.7%)	1	1	0	0
*Frontiers in Psychology*	1.4	4 (80%)	3	1	0	0
Total		23	11	7	2	3

*n*: Number of published items; AAS: Altmetric Attention Score.

**Table 5:** Top five research outputs about sport vision according to the Altimetric Attention Score (AAS).

AAS	Title	Journal/Collection Title	Publication Date (dd/mm/yyyy)	Mentions	Citations, WoS
426	Transitions between Central and Peripheral Vision Create Spatial/Temporal Distortions: A Hypothesis Concerning the Perceived Break of the Curveball	*PLoS ONE*	13/10/2010	601	37
379	Epidemiology of Sports-Related Eye Injuries in the United States	*JAMA Ophthalmology*	01/12/2016	189	16
167	Vision and Vestibular System Dysfunction Predicts Prolonged Concussion Recovery in Children	*Clinical Journal of Sport Medicine*	28/03/2018	247	37
162	What Do Football Players Look at? An Eye-Tracking Analysis of the Visual Fixations of Players in 11 v 11 Elite Football Match Play	*Frontiers in Psychology*	16/10/2020	239	0
154	Academic Difficulty and Vision Symptoms in Children with Concussion	*Optometry and Vision Science*	01/01/2018	124	19

## Supplementary Materials

The following are available online at https://www.mdpi.com/article/10.3390/ijerph18105354/s1, Figure S1: Overview of attention for the output of the five articles with the highest AAS.

## References

[1] Kim N.K., Park S.P. (2017). The Relationship between Media Sports Involvement Experiences and Sports Values and Sports Participation. Int. J. Appl. Eng. Res..

[2] Parganas P., Anagnostopoulos C., Chadwick S. (2015). ‘You’ll never tweet alone’: Managing sports brands through social media. J. Brand Manag..

[3] Nicholson M., Kerr A.A., Sherwood M. (2015). Sport and the Media: Managing the Nexus.

[4] Gilchrist P., Wheaton B., Hutchins B., Rowe D. (2013). New media technologies in lifestyle sport. Digital Media Sport: Technology, Power and Culture in the Network Society.

[5] Thorpe H. (2014). Transnational Migration and Mobilities in Action Sport Cultures.

[6] Nascimento H., Martinez-Perez C., Alvarez-Peregrina C., Sánchez-Tena M.Á. (2020). Citations Network Analysis of Vision and Sport. Int. J. Environ. Res. Public Health.

[7] Appelbaum L.G., Erickson G. (2016). Sports vision training: A review of the state-of-the-art in digital training techniques. Int. Rev. Sport Exerc. Psychol..

[8] Appelbaum L.G., Lu Y., Khanna R., Detwiler K.R. (2016). The Effects of Sports Vision Training on Sensorimotor Abilities in Collegiate Softball Athletes. Athl. Train Sports Health Care..

[9] Clark J.F., Ellis J.K., Bench J., Khoury J., Graman P. (2012). High-Performance vision training improves batting statistics for University of Cincinnati baseball players. PLoS ONE.

[10] Formenti D., Duca M., Trecroci A., Ansaldi L., Bonfanti L., Alberti G., Iodice P. (2019). Perceptual vision training in non-sport-specific context: Effect onperformance skills and cognition in young females. Sci. Rep..

[11] Schwab S., Memmert D. (2012). The Impact of a Sports Vision Training Program in Youth Field Hockey Players. J. Sports Sci. Med..

[12] Cronin B., Sugimoto C.R. (2014). Beyond Bibliometrics: Harnessing Multi-Dimensional Indicators of Performance.

[13] Priem J., Taraborelli D., Groth P., Neylon C. Altmetrics: A Manifesto. http://altmetrics.org/manifesto.

[14] Moed H.F., Glänzel W., Schmoch U. (2018). Handbook of Quantitative Science and Technology Research.

[15] Nederhof A.J. (2006). Bibliometric monitoring of research performance in the Social Sciences and the Humanities: A Review. Scientometrics.

[16] Hammarfelt B. (2014). Using altmetrics for assessing research impact in the humanities. Scientometrics.

[17] Maggio L., Meyer H., Artino A. (2017). Beyond citation rates: Real-time impact analysis of health professions education research via altmetrics. Acad. Med..

[18] Trueger N.S., Thoma B., Hsu C.H., Sullivan D., Peters L., Lin M. (2015). The Altmetric Score: A New Measure for Article-Level Dissemination and Impact. Ann. Emerg. Med..

[19] Knudson D., Kluka D.A. (1997). The impact of vision and vision training on sport performance. JPERD.

[20] Williams A.M., Ward P., Knowles J.M., Smeeton N.J. (2002). Anticipation skill in a real-world task: Measurement, training, and transfer in tennis. J. Exp. Psychol. Appl..

[21] Spera R., Belviso I., Sirico F., Palermi S., Massa B., Mazzeo F., Montesano P. (2019). Jump and balance test in judo athletes with or without visual impairments. J. Hum. Sport Exercis..

[22] Sirico F., Romano V., Sacco A.M., Belviso I., Didonna V., Nurzynska D., Castaldo C., Palermi S., Sannino G., Della Valle E. (2020). Effect of Video Observation and Motor Imagery on Simple Reaction Time in Cadet Pilots. J. Funct. Morphol. Kinesiol..

[23] Alonso Arévalo J., Cordón-Garcia J.A., Maltrás Barba B. (2016). Altmetrics: Medición de la influencia de los medios en el impacto social de la investigación. Cuad. Doc. Multimed..

[24] Shapiro A., Lu Z.L., Huang C.B., Knight E., Ennis R. (2010). Transitions between central and peripheral vision create spatial/temporal distortions: A hypothesis concerning the perceived break of the curveball. PLoS ONE.

[25] Haring R.S., Sheffield I.D., Canner J.K., Schneider E.B. (2016). Epidemiology of Sports-Related Eye Injuries in the United States. JAMA Ophthalmol..

[26] Master C.L., Master S.R., Wiebe D.J., Storey E.P., Lockyer J.E., Podolak O.E., Grady M.F. (2018). Vision and Vestibular System Dysfunction Predicts Prolonged Concussion Recovery in Children. Clin. J. Sport Med..

[27] Mack C.D., Solomon G., Covassin T., Theodore N., Cárdenas J., Sills A. (2021). Epidemiology of Concussion in the National Football League, 2015–2019. Sports Health.

[28] Aksum K.M., Magnaguagno L., Bjørndal C.T., Jordet G. (2020). What Do Football Players Look at? An Eye-Tracking Analysis of the Visual Fixations of Players in 11 v 11 Elite Football Match Play. Front. Psychol..

[29] Gallaway M., Scheiman M., Mitchell G.L. (2017). Vision Therapy for Post-Concussion Vision Disorders. Optom. Vis. Sci..

[30] Kharmalki G.W., Raizada S. (2020). Social Media Marketing in Sports: The Rise of Fan Engagement through Instagram. Soc. Mark. Sports.

