# Supplementary material for: The Role of Social Media in Sports Vision
Source: Int J Environ Res Public Health. 2021 May 18;18(10):5354. doi: 10.3390/ijerph18105354 (PMC8157247; doi:10.3390/ijerph18105354)
Supplement: Supplementary file 1 [file ijerph-18-05354-s001.zip › ijerph-1222713-supplementary.pdf]

A)

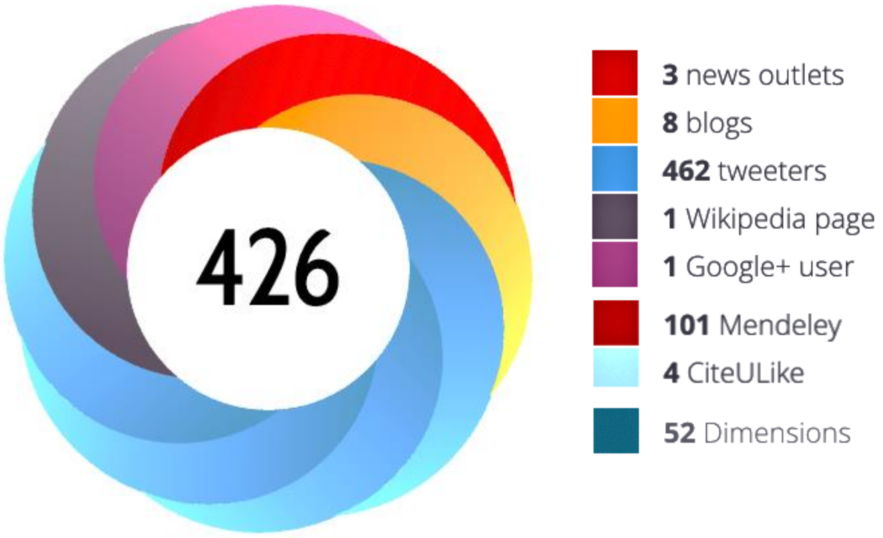
426
3 news outlets
8 blogs
462 tweeters
1 Wikipedia page
1 Google+ user
101 Mendeley
4 CiteULike
52 Dimensions

B)

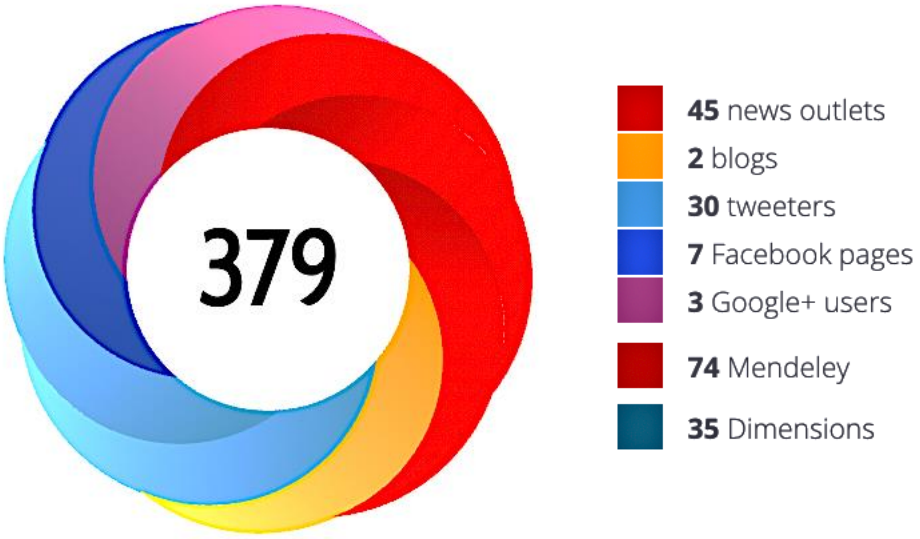
379
45 news outlets
2 blogs
30 tweeters
7 Facebook pages
3 Google+ users
74 Mendeley
35 Dimensions

C)

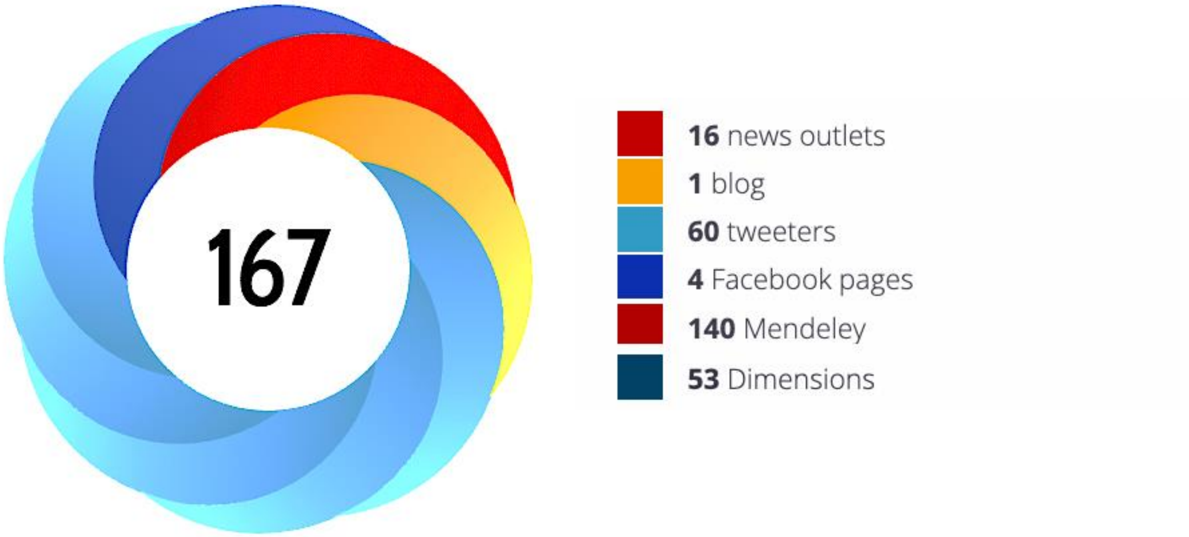
167
16 news outlets
1 blog
60 tweeters
4 Facebook pages
140 Mendeley
53 Dimensions

D)

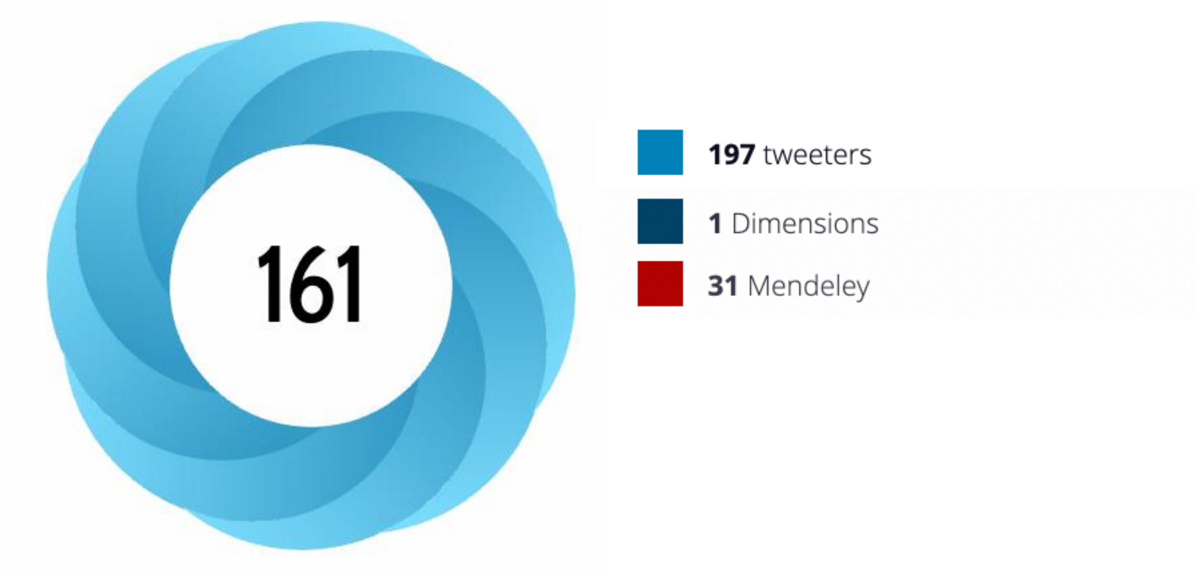
161
197 tweeters
1 Dimensions
31 Mendeley

E)

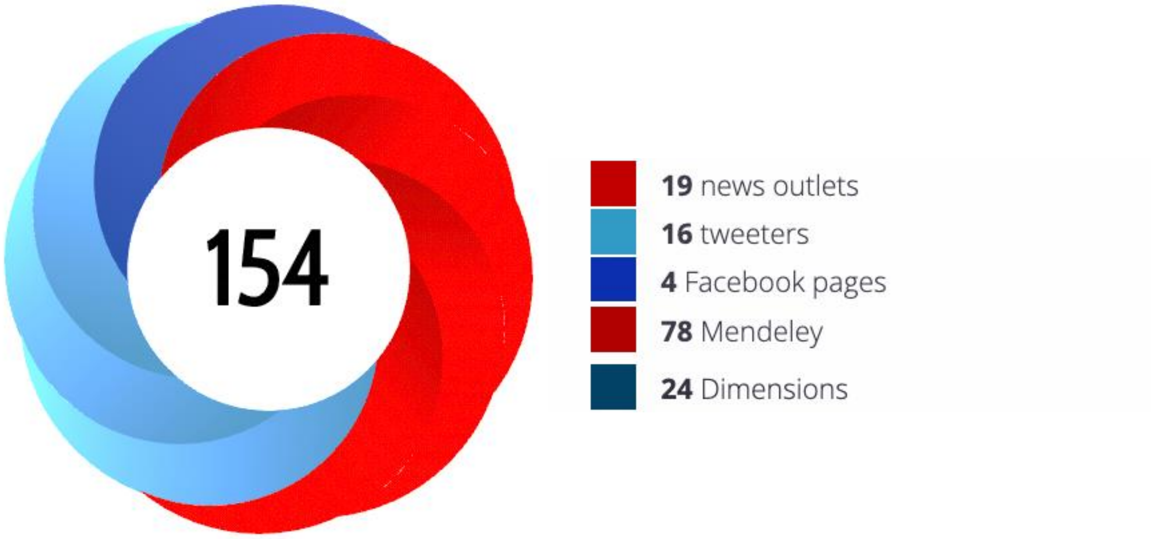

Figure S1: Overview of attention for the output of the five articles with the highest AAS. A) Transitions between Central and Peripheral Vision Create Spatial/Temporal Distortions: A Hypothesis Concerning the Perceived Break of the Curveball; B) Epidemiology of Sports-Related Eye Injuries in the United States; C) Vision and Vestibular System Dysfunction Predicts Prolonged Concussion Recovery in Children; D) What Do Football Players Look at? An Eye-Tracking Analysis of the Visual Fixations of Players in 11 v 11 Elite Football Match Play; E) Academic Difficulty and Vision Symptoms in Children with Concussion.
